# New Aspects of the Consumption Problem

**Published:** 1913-02-08

**Authors:** 


### The Hospital

The Workers' Newspaper of Administrative Medicine and Institutional Life, Administration, National Insurance and Health.

No. 1388, Vol. LIII. SATURDAY, FEBRUARY 8, 1913. PRICE ONE PENNY.

### NEW ASPECTS OF THE CONSUMPTION PROBLEM.

More than a year ago the Government of the Union of South Africa appointed a special commission to investigate consumption. Tuberculosis, within the last twenty-five years, has increased enormously at the Cape. From a national and economic aspect its importance has more than trebled since the beginning of the present century, and one side of it, silicosis or miners' phthisis, has given everyone concerned much anxious thought. So complicated and varied is the whole problem that it is not to be wondered at that in South Africa, as with us, there are widely conflicting views with regard to the best means to cope with the scourge and prevent its further spread. For that reason the decision of the authorities to investigate the matter as thoroughly as can be done by the machinery of an expert commission must be cordially welcomed, for the findings of the commission are bound to be both instructive and interesting not only to those locally concerned but to all who are interested in the problem.

We may recall that among those who tendered their views to the commissioners is Dr. Maynard, a man whose experience so far as tuberculosis is concerned is probably unequalled at the Cape, since he was for a long time Assistant Medical Officer of Health of Johannesburg and a member of the Miners' Phthisis Commission, and is at the present moment medical adviser to the largest native labour association and editor of the *Transvaal Medical Journal*. The views of such an authority, however heretical they may sound—and we confess that Dr. Maynard's evidence is the opposite of what we should expect—are at least deserving of careful and close consideration. Dr. Maynard's opinions, summarised, amount to this: He denies tuberculosis to be an infectious disease, and that close contact with a tuberculous subject materially increases the risk of healthy subjects contracting the disease; he asserts that tuberculosis is a hereditary disease, inasmuch as the liability to its more serious manifestations is inherited; and, thirdly—and this is perhaps the gravamen of his professional heresy—he insists that the decrease in the death-rate from tuberculosis, where such decrease has been proved, is due not to sanitary or hygienic improvements, but is solely an evolutionary phenomenon. As the *South African Medical Record* (to whose interesting number of September 28, which contains a full discussion of Dr. Maynard's views, we are indebted for the summary of his evidence given before the commission) rightly remarks: "These propositions amount to a categorical denial of a position taken up and acted upon by the medical profession throughout the world with an unanimity which is exceptional . . . Whatever may be the exact complexion of Dr. Maynard's destructive theories, they are about as contrary to what the profession in general believes and acts upon as one pole is distant from the other." That, we imagine, Dr. Maynard will be the first to admit, but he may also contend that he does not stand alone in holding these heterodox views. Our knowledge of the incidence of consumption, of its ætiology, pathology, and treatment is by no means exact, and though the main points on which destructive criticism of whatever views the profession holds may appear to be of minor importance, it is nevertheless a fact that our methods of treatment, especially such methods as are rightly national in the sense that they desire to cope with phthisis on a large scale, are based preeminently on data which are singularly open to objection.

We confess that we do not understand what Dr. Maynard means by an infectious disease. If tuberculosis can be caused in an individual by infecting him with the causal bacillus it is surely a splitting of hairs or Escobaritic casuistry to claim that because it cannot always be so caused it is not therefore infectious. The arguments that those exposed to infection rarely contract the disease involves us in a consideration of so many factors that little is to be gained from discussing it. Until we know more of the life-history of the bacillus within the human organism than we do at present we must be content to speculate upon the degree in which tuberculosis is infectious; but it is surely better to take it for granted, by analogy, that it is infectious and treat it as being such than to adopt a policy of negation with all its possible evils. Dr. Maynard, it seems to us, merely occupies the position previously taken up by Hueppe, Gollstein, Riffel, and the rest of the group known in Germany as Dispositionisten, and the conflict between them and those who uphold the infective-contagious view is, for the present at least, of academic interest alone. In arguing to conclusions from the primary assumption that tuberculosis is not infectious, Dr. Maynard trenches at once upon the most contentious and the most important part of the problem—the question of treatment. Here, at least, he has many of the profession with him.

So far as sanatoria are concerned, for example, the carefully considered opinion of so authoritative an expert as Grotjahn is that "a demonstrable decrease in the incidence of tuberculosis as a consequence of sanatorium treatment is not apparent, and is not to be expected in the future." The experiments of the Danish veterinary surgeon Bang have shown that segregation and isolation are not the best means of coping with the disease unless extra precautions are taken to ensure that the potentially tuberculous stock is made free from disease. To apply to humanity the principles of the Bang method as now generally followed in Germany is a method which we willingly admit may be productive of more permanent good than can be obtained from sanatoria. But the whole question of treatment needs earnest and careful consideration. Dr. Maynard's views may be heterodox, but they should at least cause the profession and the public in South Africa to think seriously before they embark on schemes of treatment so expensive and costly as those which have been tried in Germany, and which we in this country are about to initiate.

